# Detection of brain somatic mutations in focal cortical dysplasia during epilepsy presurgical workup

**DOI:** 10.1093/braincomms/fcad174

**Published:** 2023-06-01

**Authors:** Rayann Checri, Mathilde Chipaux, Sarah Ferrand-Sorbets, Emmanuel Raffo, Christine Bulteau, Sarah Dominique Rosenberg, Marion Doladilhe, Georg Dorfmüller, Homa Adle-Biassette, Sara Baldassari, Stéphanie Baulac

**Affiliations:** Sorbonne Université, Institut du Cerveau—Paris Brain Institute—ICM, Inserm, CNRS, Hôpital de la Pitié Salpêtrière, 75013, Paris, France; Department of Pediatric Neurosurgery, Rothschild Foundation Hospital EpiCARE, 75019, Paris, France; Department of Pediatric Neurosurgery, Rothschild Foundation Hospital EpiCARE, 75019, Paris, France; Department of Pediatric Neurosurgery, Rothschild Foundation Hospital EpiCARE, 75019, Paris, France; Unité de recherche 3450 DevAH, Développement, Adaptation et Handicap, Campus Brabois-Santé, Université de Lorraine, 54505, Vandoeuvre-lès-Nancy, France; Department of Pediatric Neurosurgery, Rothschild Foundation Hospital EpiCARE, 75019, Paris, France; Université de Paris Cité, MC2Lab, Institut de Psychologie, F-92100 Boulogne-Billancourt, France; Department of Pediatric Neurosurgery, Rothschild Foundation Hospital EpiCARE, 75019, Paris, France; Sorbonne Université, Institut du Cerveau—Paris Brain Institute—ICM, Inserm, CNRS, Hôpital de la Pitié Salpêtrière, 75013, Paris, France; Department of Pediatric Neurosurgery, Rothschild Foundation Hospital EpiCARE, 75019, Paris, France; Université de Paris Cité, service d’Anatomie Pathologique, APHP, Hôpital Lariboisière, DMU DREAM, UMR 1141, INSERM, 75010, Paris, France; Sorbonne Université, Institut du Cerveau—Paris Brain Institute—ICM, Inserm, CNRS, Hôpital de la Pitié Salpêtrière, 75013, Paris, France; Sorbonne Université, Institut du Cerveau—Paris Brain Institute—ICM, Inserm, CNRS, Hôpital de la Pitié Salpêtrière, 75013, Paris, France

**Keywords:** somatic mutations, focal cortical dysplasia, mTOR pathway, epilepsy surgery, stereo-EEG

## Abstract

Brain-restricted somatic variants in genes of the mechanistic target of rapamycin signalling pathway cause focal epilepsies associated with focal cortical dysplasia type II. We hypothesized that somatic variants could be identified from trace tissue adherent to explanted stereoelectroencephalography electrodes used in the presurgical epilepsy workup to localize the epileptogenic zone.

We investigated three paediatric patients with drug-resistant focal epilepsy subjected to neurosurgery. In the resected brain tissue, we identified low-level mosaic somatic mutations in *AKT3* and *DEPDC5* genes. We collected stereoelectroencephalography depth electrodes in the context of a second presurgical evaluation and identified 4/33 mutation-positive electrodes that were either located in the epileptogenic zone or at the border of the dysplasia.

We provide the proof-of-concept that somatic mutations with low levels of mosaicism can be detected from individual stereoelectroencephalography electrodes and support a link between the mutation load and the epileptic activity. Our findings emphasize future opportunities for integrating genetic testing from stereoelectroencephalography electrodes into the presurgical evaluation of refractory epilepsy patients with focal cortical dysplasia type II to improve the patients’ diagnostic journey and guide towards precision medicine.

## Teaser

Checri *et al*. report the detection of somatic mutations at low allele frequency from brain-derived trace-tissue DNA recovered from stereo-EEG electrodes used in the presurgical evaluation of patients with epileptogenic focal cortical dysplasia. This proof-of-concept study illustrates a minimally invasive approach for detecting mosaic variants in refractory epilepsies.

## Graphical Abstract

**Graphical Abstract:**
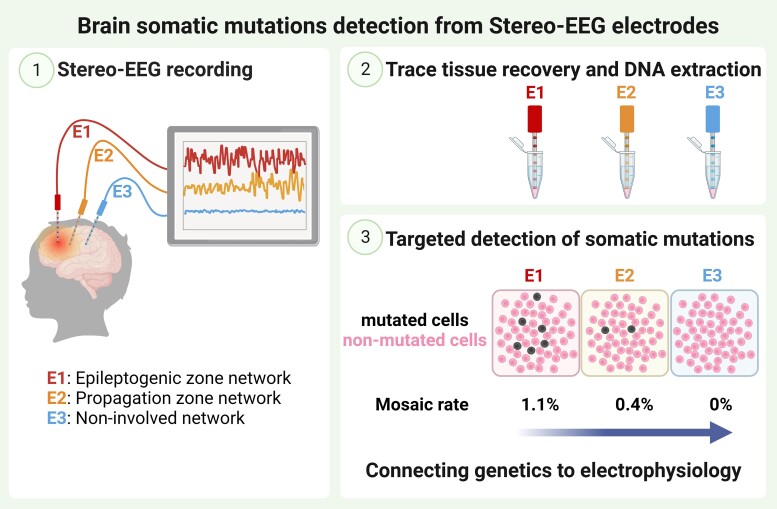

## Introduction

Focal cortical dysplasia type II (FCDII) is a type of cortical malformation manifesting with intractable epilepsy in childhood. Surgical resection of the epileptogenic zone (defined as the cortical area generating seizures) is often required to control FCDII-associated seizures.^1^

The International League Against Epilepsy (ILAE) has provided a classification system to define FCD lesions according to neuropathological findings in the resected brain specimens.^2,3^ FCDII are characterized by focal disruption of cortical cytoarchitecture and the presence of dysmorphic neurons (in FCDIIa and FCDIIb) and balloon cells (only in FCDIIb).

Postzygotic variants (or somatic variants) that arise during cortical development have emerged as important causes of FCDII.^4^ Brain somatic variants at variable levels of mosaicism have been identified in several genes (*AKT3*, *DEPDC5*, *MTOR*, *PIK3CA*, *RHEB* and *TSC1/2*) within the mechanistic target of rapamycin (mTOR) signalling pathway in individuals with FCDII.^5–12^ Yet, the genetic diagnosis depends on access to surgical brain tissue as mosaic mutations are present in only a small fraction of brain cells (mosaic level usually less than 5% in FCDII).^13^ Genetic investigation of surgical brain specimens has gained increasing interest in refractory focal epilepsy and has recently been integrated into the updated ILAE classification of FCD.^3,4^

Presurgical assessment of patients with drug-resistant focal epilepsy includes medical and neuropsychological evaluation, FDG-PET, high-resolution 3 T MRI and long-term scalp-video-EEG. In some cases, intracranial recording using stereo-EEG (SEEG) is required to accurately identify the epileptogenic zone. SEEG is an invasive technique based on the stereotactic implantation of multiple depth electrodes in the brain to record intracerebral EEG. SEEG allows anatomo-electrical correlations and tailored surgeries and may offer a therapeutic option by thermocoagulation.^14,15^ In the context of epilepsy surgery, the epileptogenic zone corresponds to the brain regions generating seizures on SEEG recordings. Recently, the regions involved in seizure production and propagation have been defined into different brain networks: epileptogenic zone network (EZN), propagation zone network (PZN) and non-involved network (NIN).^14^

Here we investigated three FCDIIa patients in which we identified somatic mutations in *AKT3* and *DEPDC5* in the DNA extracted from the resected tissue. Our findings indicate that it is possible to detect low-level somatic mutations by analysing brain-derived DNA extracted from residual tissue attached to SEEG electrodes for the presurgical evaluation of focal epilepsies associated with FCDII. We further report an association between the mutation load and the electrophysiological findings, with a higher level of mosaicism in the epileptogenic zone network.

## Materials and methods

### Patient cohort

We investigated three paediatric patients (SE1, SE2 and SE3) with refractory focal epilepsy subjected to neurosurgery at Rothschild Foundation Hospital (Paris, France), with a poor surgery outcome (Engel score Class II or III). The three patients had a neuropathological diagnosis of FCDIIa according to the ILAE classification for cortical malformations.^2,3^ A search for brain somatic variants was performed by deep-targeted panel sequencing of paired blood/brain resected tissue from the first surgery, as previously described.^6^ None of the patients were previously reported. The patients underwent SEEG in the context of presurgical evaluation for a complementary resection. An informed consent was obtained from all patients. The study protocol received approval from the ethical committee of CPP Ile de France II (*N*° ID-RCB/EUDRACT-2015-A00671-48).

### Histological analyses

Standard hematoxylin–eosin staining was performed on 4 μm formalin-fixed paraffin-embedded (FFPE) sections and 20 μm cryosections from frozen postoperative tissues. Slides were scanned in brightfield with a NanoZoomer scanner (Hamamatsu) at a 40X objective, and images were captured with the NDP.view2 software. Fluorescent immunostaining was performed according to standard procedures on 20 μm cryosections with primary antibody against SMI311 (1:500, BioLegend #837801), followed by incubation with conjugated secondary antibody (anti-mouse Alexa-555, 1:1000) and DAPI counterstain. Slides were scanned with an Axioscan (Zeiss) with a 40X objective. Whole slide scans (CZI format) were loaded on QuPath (v.0.3.2),^16^ and a pixel-trained thresholding (resolution = 0.66 µm/px, classifier = artificial neural network) was applied to measure SMI311 pixel intensity across the entire tissue section (three sections per cortex block).

### Stereoelectroencephalography sampling and analysis

Intracranial SEEG electrodes were implanted under general anaesthesia with the assistance of the robotic stereotactic assistance (ROSA) system. Intracerebral EEG was recorded using 11–14 depth electrodes (with 8–18 contacts for each electrode, 0.8 mm diameter), allowing simultaneous recordings from multiple sites. The EZN was defined by abnormal electrical discharges and dramatic changes in brain rhythms, most commonly low voltage fast discharge (LFD). The LFD may be preceded by EEG changes in the form of preictal epileptic spikes, train of spikes, or slow-wave complexes. The propagation zone network was defined by a sequential progression of abnormal neuronal activity, including the appearance of spikes or sharp waves in new brain regions, as well as changes in the frequency or amplitude of existing spikes or sharp waves. The NIN refers to the cortical network not implicated in seizures and without interictal abnormalities. SEEG-guided radiofrequency thermocoagulation (RFTC) of the epileptogenic zone network contacts was performed in patients SE1 and SE2.

### Genomic DNA extraction

Genomic DNA from trace tissue adherent to SEEG electrodes was extracted as follows: each electrode was immediately immersed in 5 ml of ice-cold PBS for cell resuspension for 24 h at 4°C. The resuspended cells/trace tissue samples were centrifuged at 3000 rpm for 5 min at 4°C, and the supernatant was removed. The samples were incubated overnight at 56°C in lysis buffer (50 mM Tris pH 7.4, 0.5% Tween-20, 200 ng/µl proteinase K) and quantified using the Qubit dsDNA high sensitivity assay kit (Thermo Fisher Scientific). Bulk DNA from the resected brain tissue was extracted as previously reported.^6^

### Droplet digital PCR

The commercial droplet digital PCR (ddPCR) mutation detection assay FAM + HEX was used to detect the *AKT3* p.E17K variant in the DNA samples from patients SE1 and SE2 with the QX200 ddPCR system (Bio-Rad Laboratories). The reactions were prepared using the ddPCR supermix for probes with 5 U of HindIII according to the manufacturer’s protocol. Data were analysed with the QuantaSoft Analysis Pro software (version 1.0.596). Wells with less than 10 000 accepted droplets were discarded. For each sample, 5 ng of genomic DNA was tested and divided into two wells (i.e. 2.5 ng of DNA per reaction) that were subsequently merged for analysis using the ‘merge wells’ function of the QuantaSoft Analysis Pro software. All experiments were performed in duplicates for statistical analyses, and variant allele frequencies (VAFs) were calculated as a mean of the fractional abundance obtained in the two duplicates. The DNA samples extracted from the bulk brain resected tissues for both SE1 and SE2 were used as mutation-positive controls. We used 20 DNA samples extracted from the blood of healthy individuals as mutation-negative controls.

### Deep-targeted amplicon sequencing

For the detection of the *DEPDC5* p.R843* variant in patient SE3, we used a deep-targeted (4000 × mean coverage) amplicon sequencing approach since no commercial ddPCR probes were available. Genomic DNA extracted from the bulk brain resected tissues of SE3 was used as a mutation-positive control. To account for possible sequencing artifacts, we used 10 DNA samples extracted from the blood of healthy individuals as mutation-negative controls. We obtained PCR amplicons (243 bp) from 10 ng of genomic DNA from mutation-positive and mutation-negative controls and 6/11 electrodes, or between 1.2 and 6.7 ng of genomic DNA for the remaining 5/11 electrodes. Libraries were run on a MiSeq sequencer (2 × 250 bp) at the iGenSeq sequencing facility at ICM. Variant calling was performed by the Data Analysis Core facility at ICM following standard procedures: reads were aligned to the hg38 reference human genome, and single nucleotide variants (SNVs) were called using the GATK pipeline and SAMtools mpileup.

### Statistical analysis

For the ddPCR sample analysis, we defined mutation-positive or mutation-negative samples based on an experimental limit of the blank (LOB) and limit of detection (LOD) values as previously described for Λ_FP_ >0.05 [Λ_FP_ corresponding to the average false-positive count, i.e. mutation-positive droplets (FAM+) in the mutation-negative control samples].^17^ Briefly, we first calculated the LOB as follows: $LOB=\Lambda_{FP}+1.645\;\sqrt{\Lambda_{FP}}+0.8$. Then, the LOD was obtained from the LOB as follows: $LOD=\;{\left(1.645+\sqrt{1.645^{2}+4\;*\;LOB}\right)}^{2}/4$. The non-integers were rounded up to the nearest integer. The electrodes presenting a FAM + droplets count above the experimental LOD were considered mutation-positive. A two-tailed *t*-test (equal variance) was used to assess if the VAF was statistically different between the SEEG-derived DNAs and the mutation-negative controls in both ddPCR and targeted deep amplicon experiments.

## Results

Three paediatric patients (SE1, SE2 and SE3) that underwent neurosurgery to treat drug-resistant focal seizures were included in this study. All three cases received a diagnosis of FCDIIa (with dysmorphic neurons) according to the ILAE criteria based on neuropathological findings.^2,3^ The first resection was considered incomplete based on the persistence of similar seizure types, abnormal postsurgical EEG and MRI. Because of persisting seizures, a new presurgical evaluation using depth SEEG recording was conducted. We hypothesized that traces of brain tissue adherent to SEEG electrodes could be used to detect somatic brain mutations.

### Patient SE1

Patient SE1 is a 3-year-old girl with drug-resistant infantile spasms and focal seizures starting at 15 days of life (**Table 1**). Surgical resection of the epileptogenic zone network in the precentral left frontal lobe was performed at 9 months, but seizures relapsed immediately after surgery (Engel III surgical outcome: worthwhile improvement, but persistence of clusters of seizures). A new presurgical evaluation that included SEEG analysis was conducted at 2.6 years of age. Eight of the 13 SEEG electrodes implanted were thermocoagulated (**Table 1**). Because of the persistence of daily seizures (although at decreased frequency after thermocoagulation), the patient underwent a functional hemispherotomy (consisting of the disconnection of the two hemispheres, with left frontal lobe cortectomy). The patient is now Engel 1 (7 months follow-up).

**Table 1:** Clinical, genetic, neuropathological and electrophysiological features

Patient	SE1	SE2	SE3
Neuropathology	FCDIIa	FCDIIa	FCDIIa
Genetic findings	AKT3 p.E17K somatic	AKT3 p.E17K somatic	DEPDC5 2-hit
Sex	Female	Male	Female
Age at seizure onset	15d	4y	6m
Age at latest follow-up	3y	12y	7y
Number of surgeries	2	3	1
Seizure types	Infantile spasms, focal seizures	Focal seizures, loss of consciousness	Focal seizures, auras
Seizure frequency	Daily	Daily	Daily
Antiseizure medications	TPM, VGB, OXC	CBZ, CLB, VPA	LTG, CLB, VPA, LEV
3 T MRI	Diffuse frontal lobe FCD	Postcentral and superior parietal FCD	Superior frontal lobe and precentral gyrus blurring
Scalp EEG (ictal)	L frontal rapid discharge	R fronto-central rapid discharge	R fronto-central rapid discharge
Stereo-EEG (ictal)	Rhythmic activation/rapid discharge in supramarginal and postcentral gyri; rapid discharge in superior frontal gyrus and motor operculum	Rapid discharges in pre- and postcentral and premotor cortex	Discharges in precentral gyrus
EZN	Pre- and postcentral gyri, premotor frontal cortex, insula, and motor operculum	Mesial central-SMA and postcentral gyrus	Precentral gyrus
Electrodes in MRI lesion	AS, FA, FS, IA, MS, OM, PA, PI, PS	FA, FS, MS, PA	LP, FA, FS
Electrodes in EZN	FA, PI, PS, IA OM	FS, MS, PA	LP, FS
Electrodes in PZN	AS, FS, IA, OM, OP, MS, PA, PP,	PA, MS, FS, OM, PA	FA, OM, PS, LP, FS
Electrodes in NIN	TA, TS, TP	AS, BL, BM, CA, FI, LS, OM, OP, PI, PP,	BL, BM, FB, FI, FM
Thermocoagulated contacts	AS3-10, FA7-16, IA2-18, MS1-7, OM1-4, PA4-6, PI1-11, PS1-7	FS1-5, MS1-5, PA1-5, PA15-18	None
Follow-up post-RFTC	NA	Electroclinical improvement for 2m	NA
SEEG-guided surgery	Yes	No	No

d, days; m, months, y, years; FU, follow-up; EZN, epileptogenic zone network; PZN, propagation zone network; NIN, non-involved network; RFTC, radiofrequency thermocoagulation; SMA, supplementary motor area; TPM, topiramate, VGB, vigabatrin, OXC, oxcarbazepine, CBZ, carbamazepine; VPA, sodium valproate; CLB, clobazam; LTG, lamotrigine; LEV, levetiracetam. Following a medication change to felbamate post-SEEG, seizure frequency was reduced in patient SE3 (1-year follow-up period).

**Figure 1:**
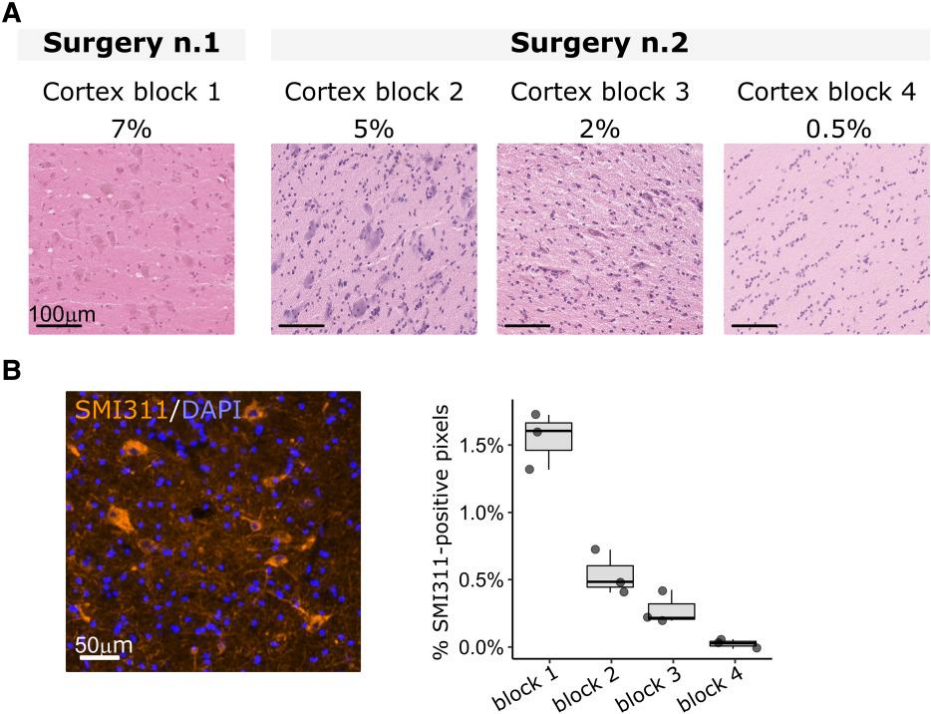
**Neuropathology and genetic findings in patient SE1.** (**A**) Hematoxylin–eosin staining on 20 µm frozen brain sections showing the variable density of dysmorphic neurons across the resected tissues with variable mutation mosaic rate from two surgeries in patient SE1. Cortex block 1: frontal-lateral cortex; Cortex block 2: premotor cortex; Cortex block 3: periventricular region; Cortex block 4: temporal cortex. (**B**) Left: representative image of SMI311-positive dysmorphic neurons (orange) in 20 µm frozen sections (DAPI-positive nuclei are indicated in blue); Right: boxplot indicating the percentage of SMI311-positive pixels in the sequenced tissue blocks (three whole sections were analysed per block). An increased percentage of SMI311-positive pixels characterized the cortex block 1 with the highest variant allele frequency.

We performed targeted panel sequencing on matched blood/brain resected tissues from the first resection and identified a brain-restricted (absent from the blood-derived DNA) recurrent variant in *AKT3* (NM_005465.7, p.E17K), with a variant allele frequency (VAF) of 7% in the fronto-lateral region (cortex block 1) (Fig. 1A). After the second surgery, we assessed the mutation distribution in three additional frozen tissue specimens by ddPCR, an ultra-sensitive and allele-specific approach for variant detection. We identified a mutation gradient from 5% in the premotor cortex (cortex block 2) to 2% in the periventricular region (cortex block 3) and 0.5% in the temporal cortex adjacent to the lesion (cortex block 4) (Fig. 1A). We performed SMI311 immunostaining, a canonical marker of dysmorphic neurons, and observed the highest signal intensity in the cortical area with the highest mosaic level (Fig. 1B).

**Figure 2:**
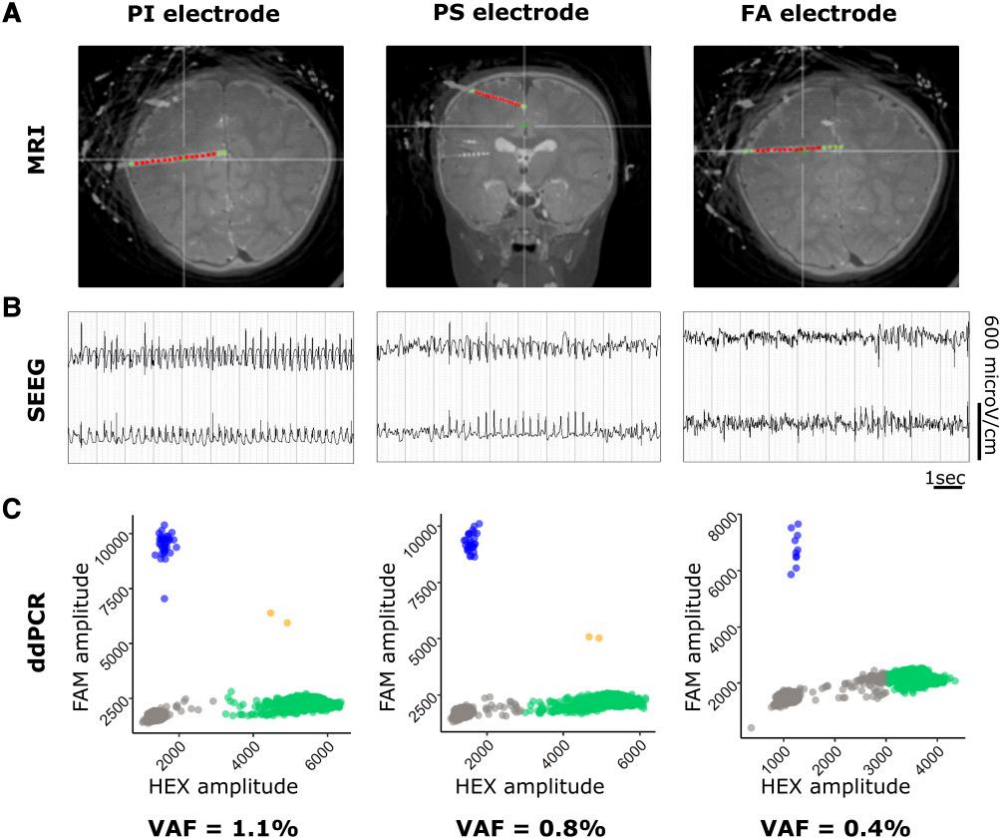
**Identification of mutation-positive SEEG electrodes in patient SE1.** (**A**) Location of mutation-positive electrodes on MRI images from patient SE1. Green plots are outside the MRI lesion; red plots are inside the lesion. (**B**) Representative SEEG traces of contacts within the epileptogenic zone network from mutation-positive electrodes. (**C**) Representative ddPCR 2D fluorescence amplitude plots showing mutant droplets of mutation-positive electrodes in patient SE1. Wild-type droplets (HEX) are indicated in green, mutant droplets (FAM) are indicated in blue, double-positive HEX/FAM droplets are indicated in orange and double-negative HEX/FAM droplets are indicated in grey. Plots were generated in R using the ggplot2 package. Fractional abundance of mutation-positive droplets (corresponding to the VAF) in mutation-negative control DNAs ranged from 0% to 0.06% (mean 0.02%, false-positive rate). FA, ascendant frontal; FAM, 6-carboxyfluorescein; HEX, 5’-hexachlorofluorescein; PI, inferior parietal; PS, superior parietal; SEEG, stereo-EEG; VAF, variant allele frequency.

We extracted the DNA from each of the 13 SEEG explanted electrodes and obtained DNA concentrations ranging from 0.2 to 2.9 ng/µl and DNA quantities ranging from 40 to 500 ng (including the eight thermocoagulated samples), indicating that the thermocoagulation procedure does not affect DNA recovery. We assessed the distribution of the *AKT3* p.E17K hotspot mutation in 9/13 electrodes by ddPCR [four electrodes were excluded due to low DNA amount (<20 ng)]. We defined an experimental LOD of *n* = 8 mutation-positive (FAM+) droplets based on mutation-negative controls (see methods). In mutation-negative controls, the average technical false-positive VAF was 0.02% (range 0–0.06%). We identified 3/9 mutation-positive electrodes (*P* < 1.12 × 10^−16^), with VAFs ranging from 0.4% to 1.1% (electrodes PI, PS and FA) (Table 2, Fig. 2). All three electrodes were in contact with the epileptogenic zone network within the premotor cortex, and located in the MRI-defined lesion. The mutation-negative electrodes were either implanted in the propagation zone network or the NIN.

**Table 2:** Genetic findings from SEEG electrodes

Electrode ID	Genomic DNA amount (ng)	Electrode location	Thermocoagulated contacts	VAF
**SE1 (*AKT3*, p.E17K)**				
PI	66	EZN, MRI lesion	Yes	1.1%
PS	208	EZN, MRI lesion	Yes	0.8%
FA	91	EZN, MRI lesion	Yes	0.4%
AS	188	PZN, MRI lesion	Yes	ns
IA	127	PZN, MRI lesion	Yes	ns
OM	501	PZN, MRI lesion	Yes	ns
MS	104	PZN, MRI lesion	Yes	ns
PA	85	PZN, MRI lesion	Yes	ns
TS	240	NIN	No	ns
SE3 (DEPDC5, p.R843*)				
LP	7.4	EZN, MRI lesion	No	ns
FS	113	EZN, MRI lesion	No	ns
CA	105	PZN	No	0.4%
OM	87	PZN	No	ns
FA	245	PZN, MRI lesion	No	ns
PS	17	PZN	No	ns
FI	7.2	NIN	No	ns
FB	157	NIN	No	ns
BL	62	NIN	No	ns
BM	5.4	NIN	No	ns
FM	33	NIN	No	ns

Electrode CA from patient SE3 was placed at the border of the MRI lesion. VAF, variant allele frequency; ddPCR, droplet digital PCR; *ns*, not significant; EZN, epileptogenic zone network; PZN, propagation zone network; NIN, non-involved network.

### Patient SE2

Patient SE2 is a 12-year-old boy with drug-resistant focal seizures since the age of 4 years. Surgical resection in the right parietal lobe was first performed at the age of 8 years, but seizures relapsed immediately after surgery (Engel III). A complementary resection was performed at 10 years without seizure control, and the patient underwent a third presurgical evaluation, including SEEG exploration. The patient had seizure improvement after thermocoagulation for 2 months (Engel 2) and is now a candidate for laser interstitial thermal therapy. We recovered brain DNA from the 14 explanted depth electrodes (among which three were thermocoagulated); we obtained DNA concentrations ranging from 0.2  to 1.2 ng/µl and DNA quantities from 1 to 44 ng.

By targeted gene panel sequencing, we identified the somatic *AKT3* (p.E17K) hotspot mutation at an ultra-low VAF of 0.25% in a small tissue specimen with few hypertrophic neurons (from the second surgery). We then searched for the mutation in the DNA extracted from 12/14 SEEG electrodes (including the three thermocoagulated, two electrodes with low DNA concentrations were excluded) but failed to detect it probably because of the ultra-low level of mosaicism (below the ddPCR sensitivity limit).

### Patient SE3

Patient SE3 is a 7-year-old girl with drug-resistant focal seizures from 6 months of age. Surgical resection in the right precentral region was performed at 6 years of age, with an Engel 2 surgical outcome (persistence of rare seizures immediately after surgery). The patient was therefore subjected to a second presurgical evaluation using SEEG recording. No thermocoagulation was performed, and following a medication change to felbamate, seizure frequency was reduced (1-year follow-up). We collected 11 individual SEEG electrodes for DNA extraction (none were thermocoagulated): we obtained between 3 and 228 ng of DNA, with a concentration ranging from 0.1 to 3.9 ng/µl per sample.

By targeted gene panel sequencing on bulk DNA from the resected brain tissue, we identified two-hit loss-of-function mutations in *DEPDC5* (NM_001242896.3): a germline heterozygous start-loss mutation c.2_6delAGATG (p.? ) and a somatic loss-of-function p.R843* variant. The germline variant was classified as ‘likely pathogenic’ based on the American College of Medical Genetics (ACMG) guidelines,^18^ and the somatic variant was classified as ‘pathogenic’ based on the recently published guidelines for pathogenicity classification of somatic alterations in tumors.^19^ The VAF of the somatic p.R843* ranged from 7% in the precentral cortex to 5% in the cingulate gyrus.

**Figure 3:**
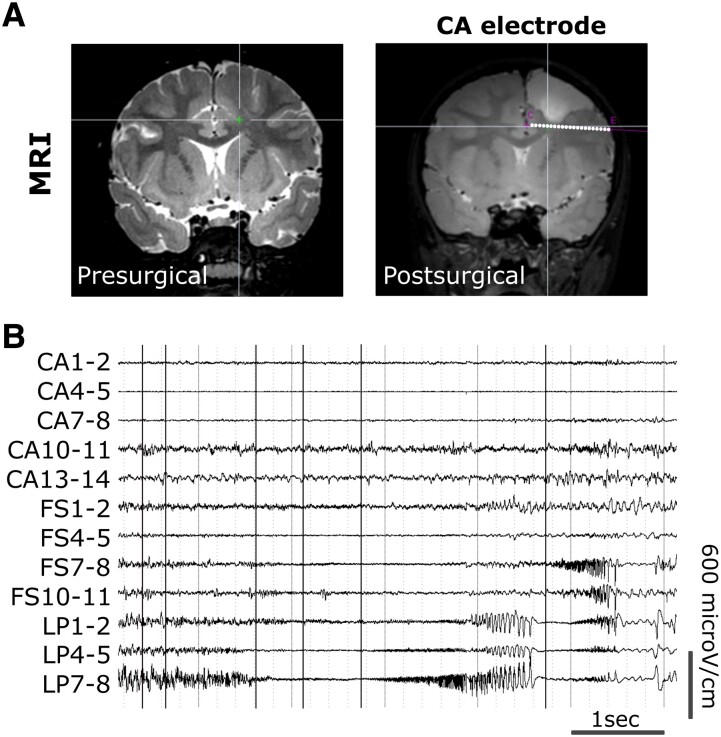
**Mutation-positive electrode (CA) in patient SE3.** (**A**) Pre- and postsurgical 3T MRI of patient SE3, showing the trajectory of the mutation-positive electrode (CA) at the bottom of the dysplastic region not resected during the first surgery (white dots). (**B**) Representative SEEG traces of the contacts within the epileptogenic zone network from LP and FS mutation-negative electrodes and the CA mutation-positive electrode in the propagation zone network. CA, anterior cingular; FS, superior frontal; LP, posterior limit.

We subsequently assessed the presence of the somatic *DEPDC5* p.R843* variant in each electrode by targeted amplicon sequencing (without genomic pre-amplification), and tested mutation-negative controls to account for possible sequencing artifacts. In mutation-negative controls, we calculated a technical false-positive VAF of 0.19% (range 0.11–0.28%). Only one electrode (CA) was considered mutation-positive, with a VAF of 0.39% (significantly above the false-positive rate, *P* = 0.002). The electrode CA was in a cortical area involved in the propagation zone network and adjacent to the border of the previously resected dysplasia (Table 2, Fig. 3). However, we did not confidently identify the *DEPDC5* somatic mutation on the two electrodes that were in the epileptogenic zone network (the VAF was below the confidence threshold).

## Discussion

Brain mosaicism is increasingly being recognized as a significant cause of drug-resistant focal epilepsy associated with FCDII.^4^ Because genetic diagnosis is hindered by direct access to brain tissue (either from surgical resection or autopsy), novel strategies have emerged to identify brain mosaic variants in biofluids. We and others provided evidence that brain variants can be detected from the circulating cell-free DNA (cfDNA) from the cerebrospinal fluid (CSF) of patients with refractory focal epilepsy.^20,21^ However, this cutting-edge approach is technically challenging due to the minimal amount of cfDNA present in the CSF. In 2019, Montier *et al*. conducted a pioneering study that identified a somatic mutation with a high VAF (16.7%) in the *MEN1* gene from the DNA extracted from trace tissues adhering to SEEG electrodes in a patient with focal epilepsy and bilateral periventricular nodular heterotopia.^22^ A second case report study identified a *KCNT1* mutation at a mosaic level of ∼25% from one patient with non-lesional focal epilepsy.^23^ Both studies were conducted on whole genome-amplified DNA and pooled SEEG electrodes.

In this study, we aimed to detect mTOR pathway somatic mutations in the context of the presurgical workup for FCDII and assess the level of mosaicism on each individual SEEG electrode. To validate this approach, we focused on three FCDIIa patients for which somatic mutations in *AKT3* and *DEPDC5* had been previously identified from the surgically resected tissues. Using targeted sequencing approaches, we detected low-level mosaic variants in brain DNA isolated from trace tissue adherent to individual SEEG electrodes from two FCDIIa patients. All mutation-positive electrodes in patient SE1 were in contact with the epileptogenic zone network and within the MRI-defined lesion, suggesting a correlation between genetics, electrophysiology, and brain pathology. This assumption is also corroborated by previous studies reporting the existence of a mutation gradient in the resected tissues, with higher mosaicism levels in the most epileptogenic area,^24–26^ and that dysmorphic neurons in FCDII tissues carry the disease-causing somatic variants.^6,26,27^ Moreover, Rampp *et al*. recently provided evidence for the contribution of dysmorphic neurons to interictal spikes, fast gamma activity and ripples, thus linking neuropathological and electrophysiological abnormalities in FCDII.^28^

This study has a limitation in its interpretation because the multiple SEEG contacts of a single electrode pass through several brain zones not always involved in the epileptogenic network. Therefore, the DNA extracted from an individual electrode could represent a combination of these different regions. As a result, the sensitivity and specificity of the study may be reduced, which could lead to false-negative results (e.g. in patient SE3 with two mutation-negative electrodes in contact with the epileptogenic zone network). Despite this ‘dilution effect’, we could detect the pathogenic mutation from the DNA extracted from the electrodes with contacts in the epileptogenic zone network.

FCD is among the most frequent malformations encountered in the paediatric epilepsy surgery population.^1^ While we here applied a targeted genetic approach to detect known variants, our work shows that genetic testing from SEEG electrode-recovered tissue can be applied to small brain lesions such as FCDII, which are caused by somatic mutations at low mosaic levels (<5%). Moreover, our findings suggest that such genetic studies could be integrated as part of the presurgical workup in FCDII to guide the localization of the resection area by targeted panel sequencing, as recently described.^9^ Since >50% of FCDII are caused by variants in mutational hotspots in *MTOR*, *PIK3CA* and *AKT3* genes, implementing presurgical genetic testing using multiplex ddPCR could also be a valuable approach, as previously showed in the resected tissues.^29,30^ On average, we extracted 60 ng of DNA from the electrodes of the epileptogenic zone network, which would be theoretically sufficient to perform capture gene panel sequencing as performed in routine settings.^6^

Currently, the resection of the epileptogenic zone is the unique therapeutic option for patients with FCD and drug-resistant epilepsy. The opportunity for a genetic diagnosis in cases that are not eligible for surgery should allow better clinical management with the use of precision-medicine therapeutic approaches, for example, targeting the mTOR signalling pathway.^31^ Integration of genetic, neuroimaging, electrophysiological or neuropathological data from patients with FCDII may also provide insights for the selection of surgical candidates and the prognostic value of the postsurgical outcome.^32–34^

This study paves the way towards integrating genetic diagnosis into the multidisciplinary epilepsy presurgical assessment by detecting mosaic mutations. Moreover, an SEEG-based approach has the potential to be applied to other conditions (with confirmed or suspected mosaic mutations) that are treated using deep brain stimulation through intracranial recording electrodes, most notably psychiatric (treatment-resistant depression and obsessive-compulsive disorder, Tourette’s syndrome) or neurologic diseases (Parkinson’s disease, genetic dystonia and tremor).^35^

## Data Availability

The data that support the findings of this study are available from the corresponding author upon reasonable request.

## Abbreviations

ACMG =: American College of Medical Genetics
ASM =: antiseizure medication
3T =: 3 Tesla
CBZ =: carbamazepine
cfDNA =: cell-free DNA
CLB =: clobazam
CZI =: Carl Zeiss image
ddPCR =: droplet digital PCR
EZN =: epileptogenic zone network
FCDII =: focal cortical dysplasia type II
FDG-PET =: fluorodeoxyglucose-positron emission tomography
FFPE =: formalin-fixed paraffin-embedded
FU =: follow-up
ILAE =: International League Against Epilepsy
LEV =: levetiracetam
LFD =: low voltage fast discharge
LOB =: limit of the blank
LOD =: limit of detection
LTG =: lamotrigine
mTOR =: mechanistic target of rapamycin
NIN =: non-involved network
OXC =: oxcarbazepine
PZN =: propagation zone network
ROSA =: robotic stereotactic assistance
RFTC =: radiofrequency thermocoagulation
SEEG =: stereo-EEG
SMA =: supplementary motor area
SNV =: single nucleotide variant
TPM =: topiramate
VAF =: variant allele frequency
VGB =: vigabatrin
VPA =: sodium valproate.
